# Impaired Left Ventricular Circumferential Midwall Systolic Performance Appears Linked to Depressed Preload, but Not Intrinsic Contractile Dysfunction or Excessive Afterload, in Paradoxical Low-Flow/Low-Gradient Severe Aortic Stenosis

**DOI:** 10.3390/jcm11102873

**Published:** 2022-05-19

**Authors:** Dorota Długosz, Andrzej Surdacki, Barbara Zawiślak, Stanisław Bartuś, Bernadeta Chyrchel

**Affiliations:** 1 Department of Cardiology and Cardiovascular Interventions, University Hospital, 2 Jakubowskiego Street, 30-688 Cracow, Poland; doris.dlugosz@gmail.com (D.D.); surdacki.andreas@gmx.net (A.S.); mbbartus@cyfronet.pl (S.B.); 2 Second Department of Cardiology, Institute of Cardiology, Jagiellonian University, 2 Jakubowskiego Street, 30-688 Cracow, Poland; 3 Intensive Care Unit, Department of Cardiology and Cardiovascular Interventions, University Hospital, 2 Jakubowskiego Street, 30-688 Cracow, Poland; zawislak.barbara@gmail.com

**Keywords:** aortic stenosis, left ventricular systolic function, afterload, preload, low-flow state

## Abstract

Paradoxical low-flow/low-gradient aortic stenosis (P-LFLG-AS) occurs in about one-third of patients with severe AS and preserved left ventricular (LV) ejection fraction (EF). Our aim was to differentiate between altered LV loading conditions and contractility as determinants of subtle LV systolic dysfunction in P-LFLG-AS. We retrospectively analyzed medical records of patients with isolated severe degenerative AS and preserved EF (30 subjects with P-LFLG-AS and 30 patients with normal-flow/high-gradient severe AS (NFHG-AS)), without relevant coexistent diseases (e.g., diabetes, coronary artery disease and chronic kidney disease) or any abnormalities which could account for a low-flow state. Patients with P-LFLG-AS and NFHG-AS did not differ in aortic valve area index and most clinical characteristics. Compared to NFHG-AS, subjects with P-LFLG-AS exhibited smaller LV end-diastolic diameter (LVd) (44 ± 5 vs. 54 ± 5 mm, *p* < 0.001) (consistent with lower LV preload) with pronounced concentric remodeling, higher valvulo-arterial impedance (3.8 ± 1.1 vs. 2.2 ± 0.5 mmHg per mL/m^2^, *p* < 0.001) and diminished systemic arterial compliance (0.45 ± 0.11 vs. 0.76 ± 0.23 mL/m^2^ per mmHg, *p* < 0.001), while circumferential end-systolic LV midwall stress (cESS), an estimate of afterload at the LV level, was similar in P-LFLG-AS and NFHG-AS (175 ± 83 vs. 198 ± 69 hPa, *p* = 0.3). LV midwall fractional shortening (mwFS) was depressed in P-LFLG-AS vs. NFHG-AS (12.3 ± 3.5 vs. 14.7 ± 2.9%, *p* = 0.006) despite similar EF (61 ± 6 vs. 59 ± 8%, *p* = 0.4). By multiple regression, the presence of P-LFLG-AS remained a significant predictor of lower mwFS compared to NFHG-AS upon adjustment for cESS (β ± SEM: −2.35 ± 0.67, *p* < 0.001); however, the significance was lost after further correction for LVd (β = −1.10 ± 0.85, *p* = 0.21). In conclusion, the association of P-LFLG-AS with a lower cESS-adjusted mwFS, an index of afterload-corrected LV circumferential systolic function at the midwall level, appears secondary to a smaller LV end-diastolic cavity size according to the Frank–Starling law. Thus, low LV preload, not intrinsic contractile dysfunction or excessive afterload, may account for impaired LV circumferential midwall systolic performance in P-LFLG-AS.

## 1. Introduction

A considerable proportion (up to 50%) of patients with severe aortic stenosis (AS) do not meet the traditional criterion of transvalvular pressure gradient [1]. Low-gradient AS with reduced LV stroke volume (SV) index may result from left ventricular (LV) systolic dysfunction (i.e., classical low-flow/low-gradient AS) [2]) or a low-flow state despite ejection fraction (EF) ≥ 50%, i.e., paradoxical low-flow/low-gradient AS (P-LFLG-AS), representing 10–25% of severe AS [2] and 25–35% of patients with preserved EF [1]. In contrast, low-gradient AS despite normal SV index is frequently associated with a less than severe AS [3]. The diagnosis of true paradoxical P-LFLG-AS, termed stage D3 symptomatic severe AS by the recent American College of Cardiology/American Heart Association (ACC/AHA) guidelines [4], is challenging and requires the elimination of possible measurement errors, corroboration of stenosis severity by multiple imaging modalities and consideration of body habitus and inadequate blood pressure control [1,2,3,4].

As shown by a meta-analysis of 18 studies including 7459 AS patients with preserved EF, mortality risk was higher in P-LFLG-AS compared to high-gradient AS and normal-flow/low-gradient AS [5]. According to the current clinical practice guidelines, aortic valve replacement (AVR), either surgical or transcatheter, should be considered in symptomatic patients with P-LFLG-AS [3,4]. Importantly, prognosis after transcatheter aortic valve implantation prognosis is worse in low-flow AS regardless of mean pressure gradient or EF [6], which strongly argues in favor of the contribution of non-valvular factors to impaired hemodynamics in low-flow AS subjects, including P-LFLG-AS. Notably, a network meta-regression analysis of 15 studies and 9737 patients with severe AS and preserved EF has demonstrated that AVR confers the least survival benefit in P-LFLG-AS compared to the remaining three flow-gradient subtypes of AS, including normal-flow/high-gradient AS (NFHG-AS) [7]. Accordingly, several potential non-valvular mechanisms, which are not ameliorated by AVR, have been implicated in the pathogenesis of decreased SV in P-LFLG-AS. These mechanisms include increased arterial load, diastolic dysfunction mediated by concentric remodeling of a small left ventricle, and intrinsic myocardial dysfunction despite preserved EF [1,2,8,9].

Our aim was to differentiate between altered LV loading conditions and contractility as determinants of subtle LV systolic dysfunction in P-LFLG-AS.

## 2. Materials and Methods

### 2.1. Patients

We retrospectively pre-screened the dataset of previously described patients with severe symptomatic degenerative AS (aortic valve area (AVA) index ≤ 0.6 cm^2^/m^2^ by the continuity equation) and EF ≥ 50% without relevant coexistent diseases and concomitant abnormalities [10,11]. Beyond more than mild aortic regurgitation or disease of another valve, significant (≥50%) epicardial coronary narrowings, a history of acute coronary syndromes or coronary revascularization [10,11], exclusion criteria included any abnormalities which could potentially contribute to low SV index: atrial fibrillation, relevant mitral stenosis, mitral insufficiency or tricuspid regurgitation and right ventricular dysfunction [1]. We also excluded subjects with diabetes or chronic kidney disease because we had previously demonstrated subtle impairment of LV contractility in AS with concomitant type 2 diabetes [12] or renal dysfunction [13].

A total of 60 patients entered the final analysis, including 30 subjects with P-LFLG-AS (SV index < 35 mL/m^2^ and mean aortic gradient < 40 mmHg) and 30 subjects with NFHG-AS (SV index ≥ 35 mL/m^2^ and mean aortic gradient ≥ 40 mmHg), corresponding to, respectively, stage D3 and D1 symptomatic severe AS, according to the current ACC/AHA guidelines on the management of valvular heart disease [4].

The institutional ethics committee approved the protocol, including the fact that informed consent was not sought as a retrospective data analysis was planned (Approval No. 122.6120.228.2016 of 27 June 2016; renewal issued on 31 January 2019).

### 2.2. Analysis of Medical Records

**Figure 1:**
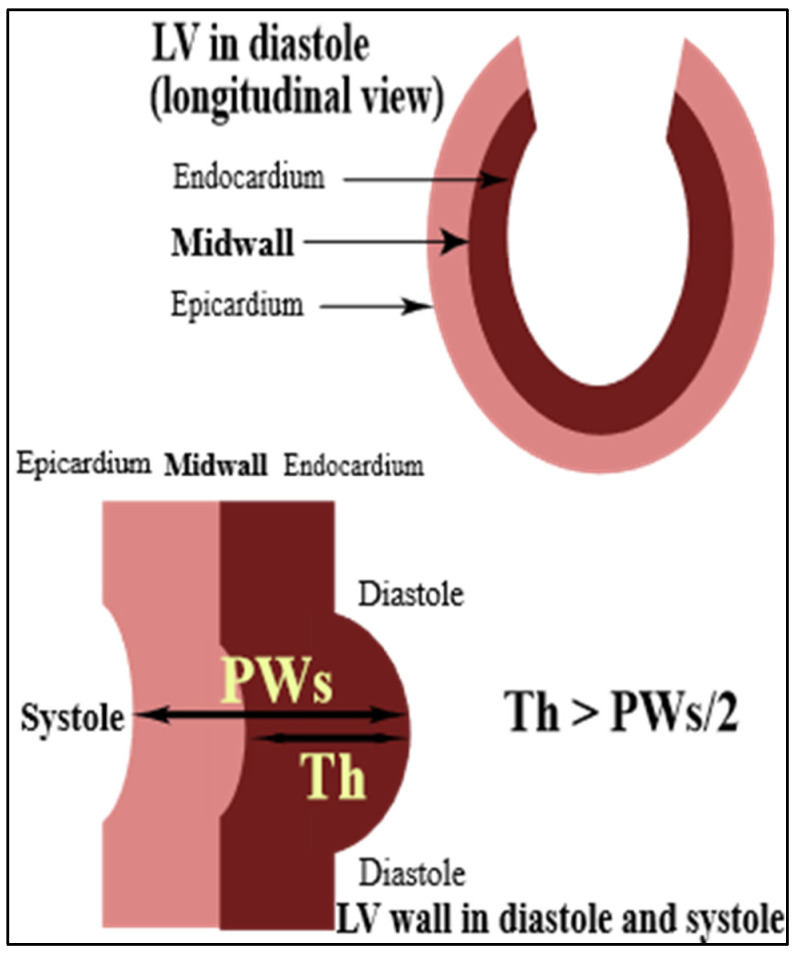
LV is represented by 2 concentric shells with the LV midwall located at the mid-point of the LV wall at end-diastole (upper panel). In systole, the LV midwall fibers migrate from the midpoint towards the epicardium due to the obvious assumption of a constant volume of the LV inner shell throughout the cardiac cycle. Consequently, Th exceeds PWs/2 (lower panel). LV: left ventricle; PWs: end-systolic LV posterior wall thickness; Th: end-systolic thickness of the LV inner myocardial shell (i.e., between the midwall and endocardium).

Routine echocardiography during the index hospitalization had been performed by an experienced sonographer. From in-hospital echocardiographic records (LV internal diameters and wall thickness) and average in-hospital cuff systolic blood pressure, we calculated LV systolic performance and circumferential systolic wall stress at the midwall level as previously described, assuming a simplified cylindrical two-shell LV model [14,15,16], similar to our earlier reports [10,11,12,13]. The model is based on a constant volume of each of two concentric shells representing the LV myocardium throughout the cardiac cycle and allows the quantification of systolic epicardial migration of a theoretical midwall fiber which is located at the midpoint of the LV wall at end-diastole [17] (Figure 1).

**Figure 2:**
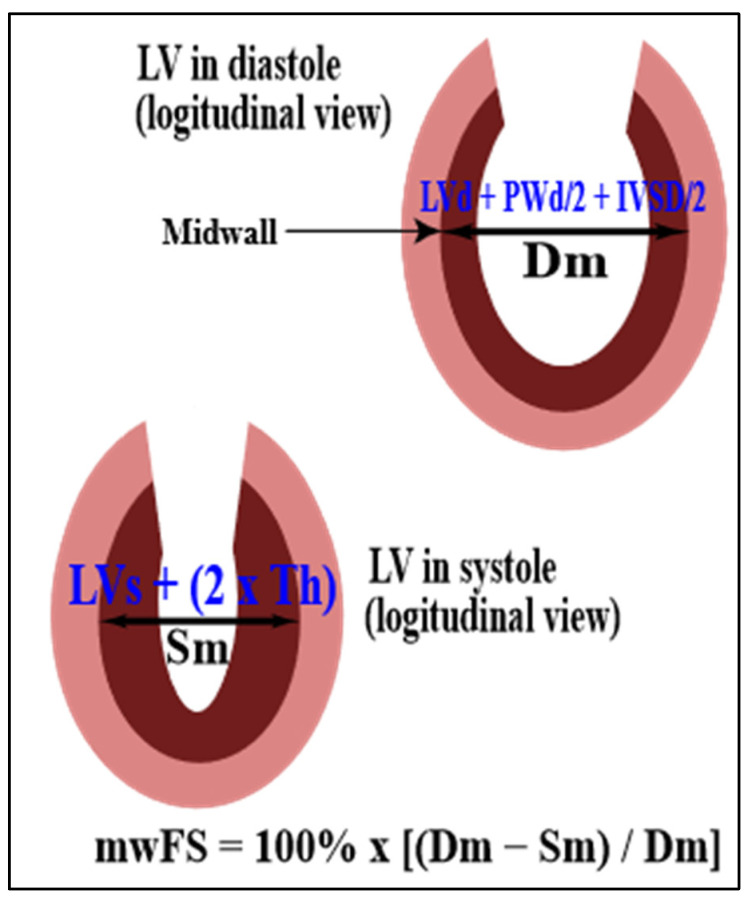
Calculation of LV midwall fractional shortening (mwFS) from LV end-diastolic diameter (Dm) and LV end-systolic diameter (Sm) at the midwall level. IVSD: interventricular septum end-diastolic thickness; LV: left ventricle; LVd: LV end-diastolic internal diameter; LVs: LV end-systolic internal diameter; PWd: end-diastolic LV posterior wall thickness; Th: end-systolic thickness of the LV inner myocardial shell (i.e., between the midwall and endocardium).

The calculation of midwall fractional shortening (mwFS) enables avoiding an overestimation of fractional shortening with conventional endocardial measurements due to radial thickening, but not shortening, of longitudinal subendocardial fibers, especially in patients with concentric LV geometry [14,15,16,17]. Accordingly, mwFS [%] was defined as follows (Figure 2):

mwFS = 100% × {[LVd + PWd/2 + IVSd/2] − [LVs + (2 × Th)]}/(LVd + PWd/2 + IVSd/2)

where LVd is end-diastolic LV internal diameter; LVs is end-systolic LV internal diameter; PWd is end-diastolic LV posterior wall thickness; IVSd is end-diastolic interventricular septum thickness; and Th is end-systolic thickness of the LV inner myocardial shell (i.e., between the midwall and endocardium), calculated on the basis of the assumption of a constant volume of the LV inner shell throughout the cardiac cycle [12,15].

**Figure 3:**
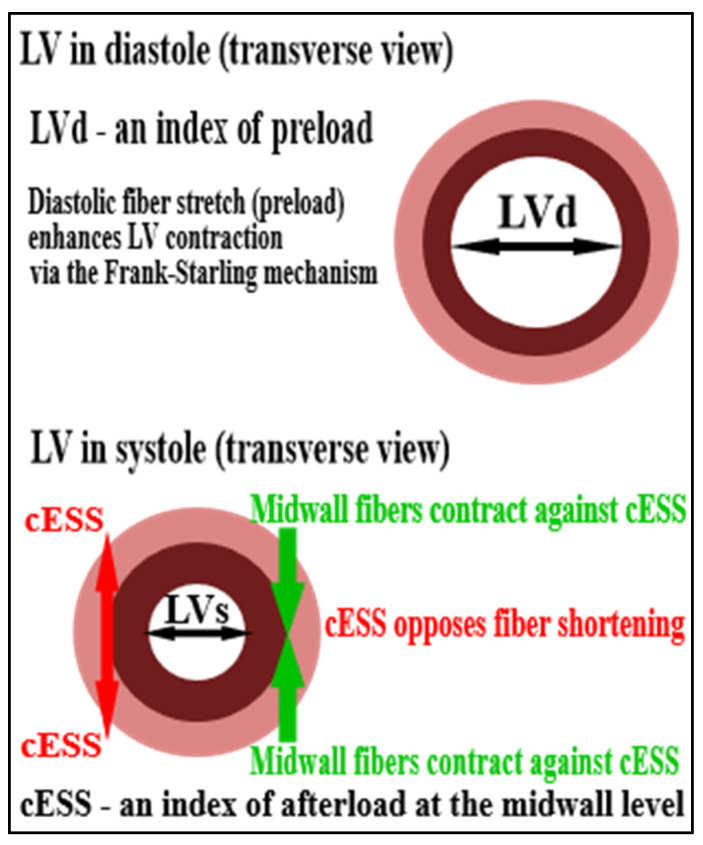
An indirect insight into load-independent intrinsic LV systolic function (i.e., contractility) by means of the analysis of relations between LV systolic performance (mwFS, LV midwall fractional shortening) and surrogate indices of LV afterload (cESS, circumferential end-systolic LV midwall stress) and preload (LVd, end-diastolic LV internal diameter).

In addition, mwFS was analyzed after adjustment for circumferential end-systolic LV midwall stress (cESS) which is directed along the same axis as systolic shortening of primarily circumferential midwall fibers, thereby providing a measure of the fundamental relationship between stress (an index of afterload) and shortening oriented in the same direction (Figure 3).

Although commonly referred to as cESS, in the present study this measure of afterload was computed taking into account not only brachial systolic pressure but also mean transaortic pressure gradient, in agreement with an approach by Carter-Storch et al. [18] and Gerdts et al. [19]. Therefore, cESS (hPa) was calculated using the following formula:

cESS = {1 + [(LVs/2 + PWs)^2^/(LVs/2 + Th)^2^]}/{[(LVs/2 + PWs)^2^ − (LVs/2)^2^]} × (LVs)^2^

× (SBP + APG_mean_) × 1.333

where PWs is end-systolic LV posterior wall thickness, SBP is cuff systolic blood pressure (mmHg), APG_mean_ is mean aortic pressure gradient (mmHg), 1.333 is a conversion factor from mmHg into hPa and all diameters are expressed in cm [16,18,19] (other abbreviations as in the previously shown formula for mwFS).

Since LV wall stress varies continuously during ejection [20,21] and these changes are pronounced in pressure overload states [21,22], this method allows adjustment for actual LV wall stress during ejection, following the original concepts of Carabello et al. [23] and Gaasch et al. [21] who plotted mean circumferential fiber shortening at the midwall and EF, respectively, against mean circumferential wall stress during shortening.

In agreement with current clinical practice guidelines [24], EF was estimated from recorded 2D images using the biplane modified Simpson’s rule and validated by one of the senior authors as previously described [12]. LV mass was calculated from M-mode measurements using the Devereux formula [24]. LV hypertrophy was defined as height-indexed LV mass >47 g/m^2.7^ in women and >50 g/m^2.7^ in men. As previously proposed, valvulo-arterial impedance (Zva) was derived from the mean aortic pressure gradient (APG_mean_), average in-hospital systolic blood pressure (SBP) and SV index using the following formula [12,25]:

Zva = (APG_mean_ + SBP)/SV index

while systemic arterial compliance (SAC) was derived from SV index and average in-hospital pulse pressure (PP) using the following equation [12,25]:

SAC = SV index/PP

### 2.3. Statistical Analysis

Data are presented as mean ± standard deviation (S.D.) or numbers and percentages. Intergroup comparisons between P-LFLG-AS and NFHG-AS were performed using the 2-tailed Student’s t-test (or Welch test in case of variance inhomogeneity according to Levene’s test) or Fisher’s test for continuous and dichotomous variables, respectively. Forward stepwise ridge regression analysis was used to address the question of whether the association of P-LFLG-AS with lower mwFS compared to NFHG-AS could be attributable to different LV loading conditions, represented by cESS and LVd reflecting, respectively, afterload and preload (Figure 3).

The study had a power of 80% to detect a mean difference in mwFS between P-LFLG-AS patients (*n* = 30) and NFHG-AS subjects (*n* = 30) of 0.74 S.D. (i.e., about 2.5% because S.D. was 3.4% pooling both groups) at a type I error rate of 0.05.

A *p*-value below 0.05 was inferred as significant. All analyses were performed by means of the Statistica 64 (data analysis software system, version 13.3.704.0; TIBCO Software Inc. (2017), Palo Alto, CA, USA).

## 3. Results

Patients with P-LFLG-AS and NFHG-AS did not differ in most clinical characteristics and AVA index (Table 1 and Table 2).

In addition to decreased SV index and mean aortic gradient, subjects with P-LFLG-AS exhibited smaller LV size (consistent with low LV preload) with pronounced concentric remodeling compared to NFHG-AS (Table 2). P-LFLG patients had higher Zva and decreased systemic arterial compliance, while cESS, an estimate of afterload at the LV level, was similar in P-LFLG-AS and NFHG-AS (Table 2). With regard to LV systolic performance, mean mwFS was depressed in P-LFLG-AS vs. NFLG-AS despite comparable EF (Table 2).

Upon forward stepwise ridge regression, the presence of P-LFLG-AS remained a significant predictor of lower mwFS compared to NFHG-AS after adjustment for cESS; however, the significance was lost after further correction for LVd (Table 3).

## 4. Discussion

Our principal finding was the lack of significant load-independent differences in mwFS, an index of LV circumferential systolic function at the midwall level, between P-LFLG-AS and NFHG-AS. In particular, the association of P-LFLG-AS with lower mwFS corrected for cESS, an index of afterload, was abolished after further adjustment for a smaller LV diastolic dimension by an average of 10 mm, a surrogate measure of decreased preload in P-LFLG-AS versus NFHG-AS.

### 4.1. Comparison with Previous Studies

Thirty years ago, in an experimental model of pressure-overload LV hypertrophy, Mirsky et al. [26] demonstrated that the correction for preload, considerably lower in animals with versus without pressure overload-induced concentric LV hypertrophy for any given afterload, entirely eliminated an artifactual depression of LV contractility derived from end-systolic stress–shortening relations when preload was not accounted for. In brief, depressed LV systolic performance might result not from an apparent impairment of the intrinsic LV contractile state, but from a smaller LVd according to the Frank–Starling law. In patients with P-LFLG-AS, this mechanism was demonstrated by Gotzmann et al. [27], who reported a decreased maximum rate of LV pressure rise during the isovolumetric contraction (dp/dt_max_), but higher dp/dt_max_ normalized for lower LV end-diastolic volume (i.e., the Starling contractile index) in P-LFLG-AS compared to NFHG-AS. That dp/dt_max_ is independent of afterload enables the separation of the effect of afterload from that of preload and intrinsic LV properties on LV performance. Notably, Eleid et al. [28] reported similar dp/dt_max_ in P-LFLG-AS and NFHG-AS; nevertheless, mean LVd was almost identical in their subjects with P-LFLG-AS and NFHG-AS. Accordingly, it can be hypothesized that a depressed mwFS at a comparable cESS in our P-LFLG-AS patients might rather be a consequence of inadequate LV preload (reflected by decreased LVd) than impaired LV contractility.

We observed similar cESS, lower cESS-adjusted mwFS (i.e., without preload correction), increased Zva and diminished systemic arterial compliance in P-LFLG-AS vs. NFHG-AS. No significant intergroup differences in cESS despite a 1.7-fold higher Zva and 1.5-fold higher systemic arterial compliance might have reflected the impact of a smaller LV size and more concentric LV geometry which counterbalanced the effect of excessive arterial load on cESS in P-LFLG-AS. An increased Zva, especially its arterial component, is a typical feature of P-LFLG-AS [1,2], confirmed in both invasive [27,28] and noninvasive [8] studies. The maintenance of cESS at a constant level over time appears pivotal for an adequate hypertrophying response to chronic LV pressure overload. Indeed, in an experimental model of LV pressure overload, Gaasch et al. [21] reported that mwFS and mean circumferential wall stress during ejection were preserved in compensated LV hypertrophy, in contrast to gradually decreasing mwFS and progressive rises of the wall stress in LV hypertrophy with concomitant pump failure. These observations were recently confirmed in an elegant longitudinal study by Ito et al. [29], who observed gradually increasing cESS and falling mwFS in patients with EF < 60% and depressed LV contractility during the progression from moderate to severe AS, while mwFS was unchanged and cESS even slightly decreased over time (by about 6%) in those with EF ≥ 60%. Additionally, in our previous retrospective observational study of nondiabetic AS subjects, we have also reported no significant differences in cESS between moderate and severe AS with EF ≥ 50% [11]. Accordingly, cESS appears to be a controlled variable in AS subjects with preserved EF, including also P-LFLG-AS.

Admittedly, we did not assess longitudinal LV systolic function in our retrospective analysis of routine echocardiographic records. However, our findings are partially consistent with earlier noninvasive studies comparing P-LFLG-AS and NFHG-AS, including novel echocardiographic techniques. In particular, circumferential LV strain [30,31,32,33] and meridional ESS [34] were comparable in P-LFLG-AS and NFHG-AS, in contrast to early impairment of longitudinal LV systolic function in P-LFLG-AS [1,30,31,32] which was linked to elevated Zva and diminished systemic arterial compliance [30,31,32,34]. Importantly, mwFS is largely controlled by circumferentially oriented fibers which predominate in the myocardial midwall, while subendocardial fibers are mainly oriented in the meridional direction [14,15,16,17,26].

Accordingly, these observations suggest a preferential impairment of LV longitudinal systolic function—not assessed in our study—in P-LFLG-AS, probably dependent on the long-time exposure of subendocardial cardiomyocytes to excessive wall stress with consequent supply–demand ischemia, apoptosis and fibrosis [34,35,36]. In particular, close associations of myocardial fibrosis with longitudinal LV dysfunction were reported in severe AS [34,37], including also P-LFLG-AS [34]. Moreover, depressed global longitudinal strain predicted adverse cardiovascular events in P-LFLG-AS [33] and moderate-to-severe AS [38,39,40,41]. Unlike LV function along the long axis, radial and circumferential systolic function may be preserved, in analogy to heart failure with preserved EF [42], which may also explain the absence of significant load-independent differences in mwFS between P-LFLG-AS and NFHG-AS in the present study. In line with this concept, compared to mwFS or circumferential strain, a decrease in longitudinal strain occurs earlier with increasing AS severity [43] or higher Zva in severe AS [44].

Additionally, it would also be interesting to analyze determinants of LV contractility in AS patients with reduced EF who had been excluded from the present study. Nevertheless, compared to patients with severe AS and normal LV contractility (by means of cESS-adjusted mwFS), those with depressed contractility and EF < 60% had similar both cESS and LVd, in contrast to AS subjects with EF ≥ 60% who exhibited lower cESS and decreased LVd (by about 7.5 mm) and concentric LV hypertrophy or remodeling in about 90% [29]. These findings suggest that reduced LV contractility can be in part attributable to low preload only in AS with preserved EF, similar to our P-LFLG-AS subjects, whose vast majority also exhibited concentric LV hypertrophy or remodeling. Finally, depressed mwFS in P-LFLG-AS could also be determined by the magnitude of diastolic dysfunction, like in hypertensive subjects [45].

Notably, these changes presumably develop before the onset of severe AS, being different in those with depressed and preserved EF. Ito et al. [29] reported an increasing proportion of subjects with high cESS (from 8% to 17%) over a 3-year interval prior to the diagnosis of severe AS with EF < 60%, especially in patients with impaired LV contractility, whose prevalence increased from 38% to 68% during the progression from moderate to severe AS. In sharp contrast, in those with EF ≥ 60%, cESS remained normal and even declined over time, which was more pronounced in the case of reduced LV contractility. Importantly, in AS with EF ≥ 60%, low LV contractility was present already in 17% of patients with moderate AS, further increasing to 24% during the progression to severe AS [29].

### 4.2. Clinical Implications

These results highlight the relevance of avoiding excessive diuretic use and preventing bradycardia in order to optimize stroke volume and maintain adequate cardiac output in P-LFLG-AS.

The dependence of depressed LV performance on low preload in P-LFLG-AS supports the concept of so-called preload stress echocardiography. P-LFLG-AS poses a diagnostic challenge because this condition requires multimodal imaging to differentiate true severe AS from pseudosevere AS, present in about one-third of patients with apparent P-LFLG-AS [1,46]. In asymptomatic or equivocal symptomatic subjects with LFLG-AS, Kusunose et al. [47] reported the ability of preload augmentation by leg positive pressure to increase transvalvular flow rate by an average of 11%, which enabled the confirmation of true severe AS in 14 out of 32 P-LFLG-AS cases by a projected AVA at a normal mean transvalvular flow rate of 250 mL/s. Dobutamine stress echocardiography, recommended in classical LFLG-AS (i.e., with reduced EF), may also be considered in P-LFLG-AS with uncertain diagnosis [48]. Nevertheless, since dobutamine can induce adverse side effects, e.g., arrhythmias and hypotension, in P-LFLG-AS subjects with restrictive LV physiology, preload stress challenge could be a potential alternative in P-LFLG-AS with an adequate preload reserve, i.e., a rise in transvalvular flow by at least 15% in response to preload recruitment, which is necessary to calculate a projected AVA [48].

The prognostic relevance of advanced indices of LV systolic performance is of clinical relevance. Depressed LV longitudinal strain was associated with adverse clinical outcome in AS [33,38,39,40,41] and optimally treated dilated cardiomyopathy [49], providing incremental prognostic value to EF. With regard to mwFS, a measure of circumferential LV systolic function, reduced cESS-adjusted mwFS predicted mortality in severe AS and EF ≥ 60% but not EF < 60% [29]. In addition, combined circumferential and longitudinal LV was related to aortic valve-dependent events in asymptomatic severe AS beyond traditional risk predictors [50], whereas new-onset low mwFS predicted CV death and heart failure hospitalizations independently of time-dependent EF changes in asymptomatic mild or moderate AS participating in the SEAS study [51]. In addition, midwall LV replacement fibrosis by means of late gadolinium enhancement coincided with lower EF [52], was independently associated with mortality in moderate or severe AS [52,53] and did not resolve after AVR [54]. Therefore, it might be speculated that extended imaging of LV function and structure by both advanced echocardiography and magnetic resonance might be helpful in the future optimization of the timing of interventional treatment in AS.

### 4.3. Limitations of the Study

First, as our retrospective study was based on measurements performed on pre-recorded echocardiographic images from the index hospitalization, we had no opportunity to analyze LV systolic function along the longitudinal axis or to apply other advanced echocardiographic techniques. Notably, impaired longitudinal LV performance precedes circumferential dysfunction in AS [34,37,43,44]. On the other hand, we aimed to differentiate the impact of LV loading conditions and intrinsic myocardial dysfunction on LV performance. An analysis of the circumferential stress–shortening relationship appears more suitable for this purpose because Aurigemma et al. [16] demonstrated that long-axis shortening was unrelated to meridional ESS, in contrast to the inverse mwFS–cESS relationship, which suggests that factors other than afterload considerably affect longitudinal systolic function. Second, we assumed LVd as an approximate measure of preload, in contrast to experimental studies [17,26] where preload was represented by LV end-diastolic wall stress. However, LV end-diastolic pressure (LVEDP) was unknown in our study subjects. Nevertheless, Peverill [55] has recently discussed limitations inherent in various clinical definitions of preload, considered as the stimulus that directly activates the Frank–Starling mechanism, including LV volume/diameter, LVEDP and LV end-diastolic wall stress. Additionally, the proportion of patients on diuretics, known to decrease LV filling pressure, was similar in P-LFLG-AS and NFHG-AS. Third, the lack of an echocardiographic core lab poses another limitation to the study. Nonetheless, both image acquisition and measurements were performed by experienced sonographers. Fourth, medical records of consecutive AS patients with a variety of coexistent diseases were pre-screened. However, in order to limit the heterogeneity of the study group, we excluded from the final analysis the subjects with diabetes, chronic kidney disease, significant coronary artery disease, more than mild aortic insufficiency or disease of another valve, as well as with any abnormalities which could potentially contribute to a low-flow state. Fifth, a low number of patients who entered the final analysis poses a considerable limitation of the study. Finally, the potential effects of cardiovascular drugs on LV function cannot be excluded. Nevertheless, the proportion of study subjects on renin–angiotensin axis antagonists, beta-blockers and calcium channel blockers was similar among patients with P-LFLG-AS and NFHG-AS.

## 5. Conclusions

The association of P-LFLG-AS with lower cESS-adjusted mwFS, an index of afterload-corrected LV circumferential systolic function at the midwall level, appears secondary to a smaller LV end-diastolic cavity size according to the Frank–Starling law. Thus, low LV preload, not intrinsic contractile dysfunction or excessive afterload, may account for impaired LV circumferential midwall systolic performance in P-LFLG-AS.

## Figures and Tables

**Table 1:** Clinical characteristics of patients with P-LFLG-AS vs. NFHG-AS.

Characteristic	P-LFLG-AS *n* = 30	NFHG-AS *n* = 30	*p*-Value ^a^
Age, years	69 ± 11	70 ± 10	NS
Women/men, *n*	18/12	16/14	NS
Hypertension, *n* (%)	25 (83%)	24 (80%)	NS
Body mass index, kg/m^2^	29.0 ± 4.2	28.4 ± 4.5	NS
eGFR, mL/min/1.73 m^2^	75 ± 13	77 ± 15	NS
Systolic blood pressure, mmHg	136 ± 14	129 ± 17	NS
Diastolic blood pressure, mmHg	73 ± 8	66 ± 8	**0.02**
Medication, *n* (%)			
ACEI or ARB	12 (40%)	10 (33%)	NS
Beta-blockers	16 (53%)	15 (50%)	NS
Diuretics	13 (43%)	12 (40%)	NS
Calcium-channel blocker	10 (33%)	11 (37%)	NS

Data are presented as mean ± standard deviation or numbers (percentages). ^a^ *p*-values below 0.05 are denoted in bold. ACEI: angiotensin-converting enzyme inhibitor; ARB: angiotensin receptor blocker; eGFR: estimated glomerular filtration rate according to the CKD-EPI formula; NFHG-AS: normal-flow/high-gradient severe aortic stenosis; NS: non-significant; P-LFLG-AS: paradoxical low-flow/low-gradient severe aortic stenosis.

**Table 2:** Hemodynamic characteristics of patients with P-LFLG-AS vs. NFHG-AS.

Characteristic	P-LFLG-AS *n* = 30	NFHG-AS *n* = 30	*p*-Value ^a^
AVA index, cm^2^/m^2^	0.4 ± 0.1	0.4 ± 0.1	NS
Mean aortic pressure gradient, mmHg	31 ± 8	54 ± 13	**<0.001**
LV end-diastolic diameter, mm	44 ± 5	54 ± 5	**<0.001**
LV end-systolic diameter, mm	28 ± 7	34 ± 7	**<0.001**
End-diastolic LV posterior wall thickness, mm	12 ± 2	12 ± 2	NS
End-diastolic interventricular septum thickness, mm	14 ± 4	14 ± 3	NS
LV mass index, g/m^2.7^	60 ± 20	111 ± 161	0.1
LV hypertrophy, *n* (%)	20 (67%)	28 (93%)	**0.02**
Relative LV wall thickness	0.62 ± 0.16	0.49 ± 0.07	**<0.001**
EF, %	61 ± 6	59 ± 8	NS
Stroke volume index, mL/m^2^	27.6 ± 4.5	45.7 ± 9.1	**<0.001**
LV midwall fractional shortening, %	12.3 ± 3.5	14.7 ± 2.9	**0.006**
Circumferential end-systolic LV midwall stress, hPa	175 ± 83	198 ± 69	NS
Valvulo-arterial impedance, mmHg per mL/m^2^	3.8 ± 1.1	2.2 ± 0.5	**<0.001**
Systemic arterial compliance, mL/m^2^ per mmHg	0.45 ± 0.11	0.76 ± 0.23	**<0.001**

Data are presented as mean ± standard deviation or numbers (%). ^a^ *p*-values below 0.05 are denoted in bold. AVA: aortic valve area; EF: LV ejection fraction; LV: left ventricular; other abbreviations as in Table 1.

**Table 3:** Forward stepwise ridge regression analysis of predictors of LV midwall fractional shortening in patients with P-LFLG-AS compared to NFHG-AS adjusted for estimates of LV afterload (cESS) and preload (LVd).

Predictors of LV Midwall Fractional Shortening	Nonstandardized Regression Coefficient ± SEM	*p*-Value ^a^
**Unadjusted for either cESS or LVd** (*R*^2^ = 0.12, *p* = 0.008)		
P-LFLG-AS vs. NFHG-AS	−1.95 ± 0.79	**0.015**
AVA index, per decrease by 0.1 cm^2^/m^2^	−0.67 ± 0.40	0.09
**Adjusted for cESS** (*R*^2^ = 0.38, *p* < 0.001)		
P-LFLG-AS vs. NFHG-AS	−2.35 ± 0.67	**<0.001**
cESS, per increment by 20 hPa	−0.41 ± 0.08	**<0.001**
AVA index, per decrease by 0.1 cm^2^/m^2^	−0.56 ± 0.34	0.10
**Adjusted for cESS and LVd** (*R*^2^ = 0.42, *p* < 0.001)		
P-LFLG-AS vs. NFHG-AS	−1.10 ± 0.85	0.21
cESS, per increment by 20 hPa	−0.47 ± 0.09	**<0.001**
LVd, per decrease by 5 mm	−0.71 ± 0.31	**0.03**
AVA index, per decrease by 0.1 cm^2^/m^2^	0.55 ± 0.33	0.10

^a^ *p*-values below 0.05 are denoted in bold. cESS: circumferential end-systolic LV midwall stress; LVd: LV end-diastolic diameter; SEM: standard error of the mean; *R*^2^: adjusted coefficient of multiple determination; other abbreviations as in Table 1 and Table 2.

## Data Availability

The data presented in this study are available on request from the corresponding author.
